# *Bifidobacterium* modulation of tumor immunotherapy and its mechanism

**DOI:** 10.1007/s00262-024-03665-x

**Published:** 2024-04-02

**Authors:** Bo Pei, Shixuan Peng, Chuying Huang, Fuxiang Zhou

**Affiliations:** 1 Hubei Key Laboratory of Tumor Biological Behaviors, Department of Radiation Oncology and Medical Oncology, Zhongnan Hospital of Wuhan University, Hubei Cancer Clinical Study Center, Wuhan, China; 2 Department of Oncology, The Central Hospital of Enshi Tujia and Miao Autonomous Prefecture, Enshi Clinical College of Wuhan University, Enshi, China; 3 Department of Oncology, Graduate Collaborative Training Base of The First People’s Hospital of Xiangtan City, Hengyang Medical School, University of South China, Hengyang, China; 4 Hubei Selenium and Human Health Institute, The Central Hospital of Enshi Tujia and Miao Autonomous Prefecture, Enshi, China; 5 Hubei Provincial Key Laboratory of Selenium Resources and Bioapplications, Enshi, China

**Keywords:** *Bifidobacterium*, Immunotherapy, Immune checkpoint inhibitors, Gut probiotics, Mechanism

## Abstract

The advent of tumor immunotherapy in patients has revolutionized the treatment of tumors and significantly improved survival rates for a wide range of tumors. However, the full therapeutic potential of immune checkpoint inhibitors (ICIs) has yet to be realized, as not all patients have a lasting survival benefit from them, and a significant proportion of patients show primary or acquired resistance to immunotherapy. *Bifidobacterium* is one of the most common probiotics, and its antitumor and immunomodulatory effects have been demonstrated in recent years, but its immunomodulatory effects in tumors, especially on ICIs and in combination, have not been extensively studied in clinical practice, and its effects on the immune system and the mechanisms that modulate immunotherapy are largely unknown. Therefore, this review will focus on the immunomodulatory effects of Bifidobacteria in malignancies and the possible mechanisms of action of Bifidobacteria on immunotherapy in the hope of providing a basis for further research and better application of Bifidobacteria in clinical practice.

## Introduction

Cancer is one of the world’s deadliest diseases, with morbidity and mortality rates increasing rapidly [1, 2]. In both men and women, cancer is the primary killer between the ages of 40 and 79 for both genders, accounting for 21% of fatalities worldwide, and between the ages of 60 and 79, cancer is the top killer [3, 4]. Other studies proved that many patients are less likely to receive guideline-consistent care, even when patient comorbidities are taken into account [5–7]. In addition, many patients are not only less likely to undergo surgery, but often rarely receive invasive treatments, as most cancer-directed therapies do not extend survival beyond the potential adverse effects and impact on quality of life, and so are not suitable for some patients [8, 9]. Of these, the third most prevalent cancer diagnosed worldwide is colorectal cancer, and its incidence is rising in low- and middle-income countries [10].

Although a variety of treatments have been applied to cancer over the past few decades, including surgery, chemotherapy, radiotherapy and targeted therapy, the prognosis remains unsatisfactory [10]. Fortunately, the advent of tumor immunotherapy has revolutionized the treatment of tumors [11]. Tumor immunotherapy refers to the application of immunological principles and methods to specifically remove small residual tumor lesions, inhibit tumor growth, and break immune tolerance by activating immune cells in the body and enhancing the body’s anti-tumor immune response. This approach has been applied to the treatment of a variety of tumors. Specifically, immunotherapy has significantly improved survival rates for many types of tumors. Several clinical studies are currently underway, primarily in patients with metastatic melanoma, to assess the potential of probiotics transplantation to enhance immune checkpoint blockade therapy. Clinical trials are also underway for prostate cancer, gastrointestinal cancer, and mesothelioma. Researchers combining active bacteria and immunotherapy have found to enhance the anti-tumor response and improve survival in patients with metastatic renal cell carcinoma, a finding that suggests that modulating gut bacteria can enhance the potential of immunotherapy for cancer patients [12–18].

As advances in genetic engineering, medicine, and biology have increased the availability and efficacy of bacteria in the treatment of tumors, gut probiotics modulation can influence the host’s response to many types of cancer therapy, particularly immunotherapy [13, 17]. Immunotherapy is receiving increasing attention and is becoming a supportive tool for oncology treatment [19]. Bifidobacteria have a role in the prevention of intestinal cancer in healthy intestinal microbiota by influencing intestinal probiotics metabolism and enhancing the host immune response; adhering to and degrading potential carcinogens; altering intestinal probiotics; producing anti-cancer and anti-mutagenic substances; promoting intestinal motility and enhancing the host immune response; and influencing host physiological activity [20–22].

Probiotics can mediate the regulation of the growth of intestinal bacteria and fungi [23], adhering to the surface of the gastrointestinal tract, aiding in maintaining the host’s intestinal microbial balance [24, 25]. Directly or indirectly affecting the colonization and growth of bacteria, regulating cell proliferation, the immunoregulatory mechanisms of probiotics mainly include mucosal barrier function, enhanced phagocytic activity of macrophages, and promotion of IgA production [26]. Probiotics can regulate the host’s immune function through the production of enhanced intestinal mucosal barrier function, inhibition of pathogen growth and adhesion, regulation of immune cell activity, and promotion of immune factor production [27]. Probiotic intervention can change the diversity of intestinal microbiota and regulate the abundance level of microbial communities, demonstrating inhibitory effects on the growth of harmful microorganisms in the intestine, particularly the beneficial appearance of intestinal health-promoting bacteria, reflecting the physiological effects of the probiotic preparation. Previous studies have reported how microbial communities promote the recruitment of T cells in mouse organs [28]. Regarding the immune therapy of intestinal microorganisms and tumors, recent studies have reported related human data. In different categories of epithelial tumors (non-small cell lung cancer, renal cell carcinoma, urothelial carcinoma), and malignant melanoma, certain intestinal microbial communities have predictive effects on the efficacy of immunotherapy. The phenotypes of responders and non-responders in immune response can be altered through microbial transplantation, with germ-free mice or mice pre-treated with antibiotics obtaining corresponding phenotypes after the transplantation of the fecal microbial community of responders or non-responders. Feeding specific strains of bacteria can allow non-responding mice to regain the responding phenotype [29–31]. Bifidobacteria has been shown to positively induce an anti-tumor immune response [32]. Researchers are becoming aware that the intestinal probiotics and the host are in a mutually beneficial symbiosis, and the intestinal probiotics can influence the state of the host’s immune system by interacting with the lymphoid tissues associated with the intestinal mucosa, promoting and regulating both innate and adaptive immunity [33].

Immune checkpoint inhibitors (ICIs) act on immune checkpoints to enhance the immune response or relieve immunosuppression. Recent research has demonstrated that immune checkpoint inhibitor medication is significantly influenced by gut probiotics [34]. Since not all patients have a durable survival advantage from ICIs and a sizable portion of patients exhibit primary or acquired immunotherapy resistance, the full therapeutic potential of ICIs is still not fully realized [35]. For instance, up to 50% of melanoma patients and 25–44% of non-small cell lung cancer patients have primary ICIs resistance [36–38]. Exploring novel approaches to enhance treatment response and overcome resistance to this potent medication presents a significant challenge in light of this. The gut microbiota plays crucial roles in the body, including those related to intestinal immunity, controlling the levels of secondary bile acids, and influencing the metabolites made in the gut [39, 40]. Imbalances in the gut microbiota may promote the development of a variety of local and systemic diseases and affect the efficacy of treatment. Therefore, appropriate modulation of the gut probiotics may help to prevent the progression and development of tumors, and it may be beneficial in supporting effective treatment. The makeup of the gut microbiota may be altered therapeutically by the use of fecal microflora transplantation (FMT), probiotics, prebiotics, and synbiotics, among other methods [39].

Probiotics are currently the most often employed substances to alter the intestinal probiotics in a number of situations. One of the most widely utilized probiotic ingredients is *Bifidobacterium*. Despite extensive research into *Bifidobacterium*’s probiotic abilities, its immunomodulatory role in tumors has not been extensively investigated [22, 41, 42]. The immunomodulatory effects of tumors have not been thoroughly studied, and it is unclear how much of an effect they have on the immune system. Based on the current state of research, synergistic development of basic and clinical research for clinical translation allows for effective translation of preclinical research models of immunotherapy into clinical application. In this review, we will discuss the immunomodulatory role of *Bifidobacterium* in malignancies and then describe the possible mechanisms of *Bifidobacterium* for immunotherapy, with the expectation of providing a basis for further research on *Bifidobacterium* and better applications in clinical practice.

## Advances in preclinical and clinical studies of *Bifidobacterium*-modulatory immunotherapy

**Fig. 1:**
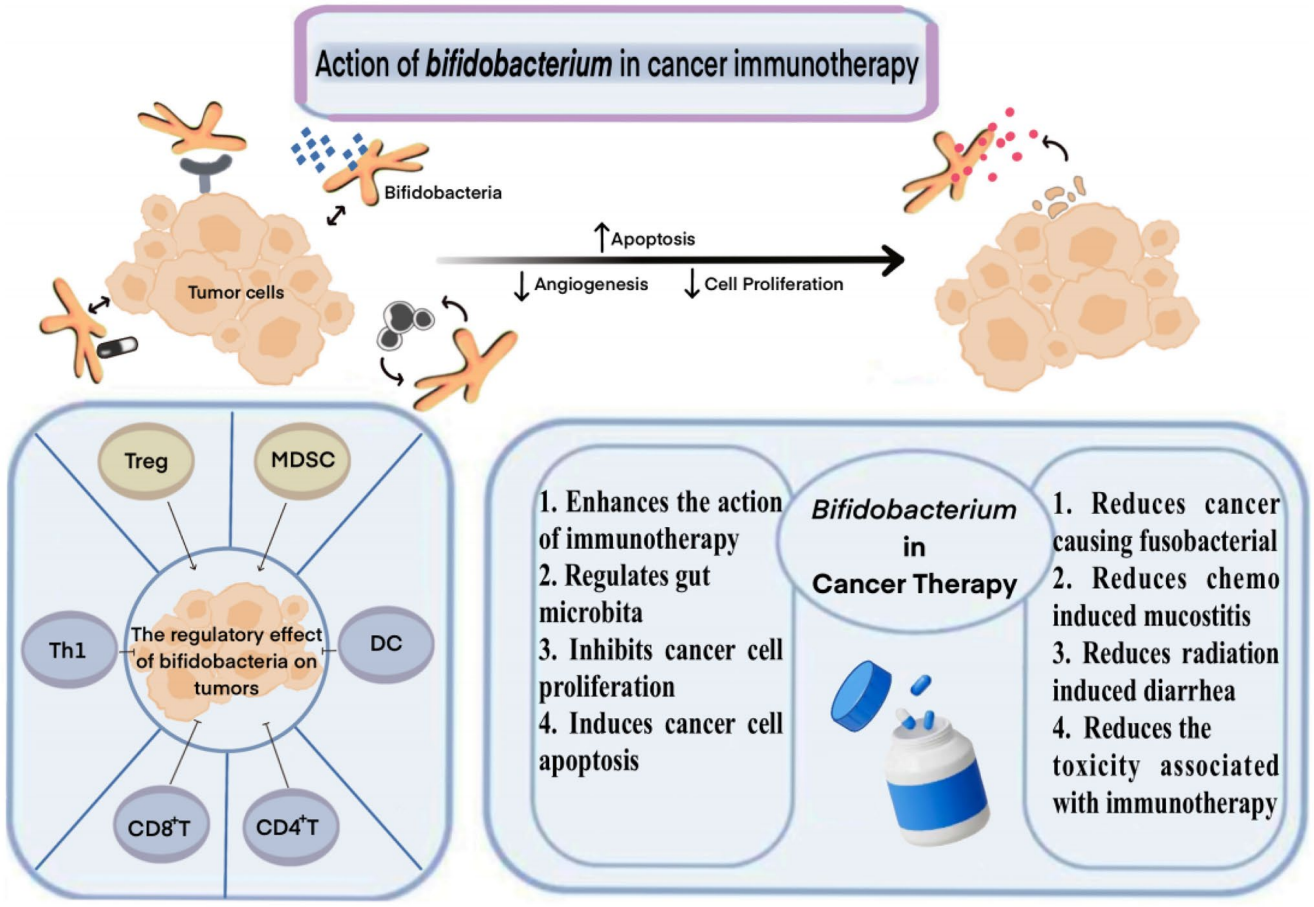
*Bifidobacterium*-modulatory cancer immunotherapy

*Bifidobacterium* colonies are small, smooth, convexly rounded, with intact margins, and creamy to white in color and play a significant role in the maturation of the immune system [43–46]. Bifidobacteria have been shown to have significant antitumor effects. Dynamic monitoring of fecal bacterial levels in tumor-bearing mice using 16S ribosomal RNA gene sequencing revealed that *Bifidobacterium* significantly delayed the growth of tumors in mice [47]. *Bifidobacterium* exerts this effect in three main ways: First, the immune system is activated by *Bifidobacterium*, which is consistent with its immunological-enhancing role. Second, it affects the gut probiotics biochemical metabolism, which reduces the production of carcinogens. Finally, *Bifidobacterium* has the ability to cause apoptosis in tumor cells, maintaining a normal cell phenotype and halting the development of tumors [48–52] (Fig. 1).

A balanced symbiotic microbiota is necessary for an appropriate response to tumor therapy, according to studies. This microbiota’s control over the action of myeloid-derived cells in the TME is what makes this therapy effective [53]. Immune checkpoint inhibitors, which start an efficient tumor attack by disabling the body’s immune system’s safety mechanism, represent a significant advance in the treatment of tumors [54, 55]. Intestinal probiotics significantly enhance anti-tumor CD8^+^ T-cell immunity, especially *Bifidobacterium* [32].

Given the possible synergy between Bifidobacteria and immunotherapy, a number of animal model studies and preclinical studies related to this area have emerged in recent years. For example, by contrasting the diversity and makeup of the fecal microbiota in responders and non-responders, one researcher looked at the relationship between the gut microbiota and treatment response in patients receiving PD-1/PD-L1 blocking, and although the specific bacterial species required for the effects of a PD-1 blockade have not been identified, an acceptable response to anti-PD-1 treatment was substantially correlated with the presence of microorganisms from the species *Bifidobacterium* [48]. Sivan et al. explored the difference in efficacy between two mouse models of melanoma (JAX/TAC) with different intestinal commensal probiotics treated with antibodies targeting PD-L1 (*α*PD-L1 mAb) and found that *Bifidobacterium* spp. were significantly increased in JAX mice with slow tumor growth and a better response to PD-L1 blockade treatment [47]. The antitumor effectiveness of PD-L1 blockade was improved by oral delivery of probiotics, may be able to induce positive antitumor immune effects [55]. *B. pseudolongum* monoculture colonization in combination with ICIs treatment significantly enhanced splenic Th1 cell activation and effector function compared to Colidextribacter control colonized mice, as evidenced by IFN-*γ* production. *B. pseudolongum* induces Th1 differentiation and activates Th1 effector T cells together with CTLA-4 antibody [56]. A study by Gopalakrishnan et al. [57] found that *Bifidobacterium bifidum* may boost anti-tumor immunity and increase the effectiveness of anti-PD-L1 treatment in a mouse model of melanoma. In addition, *Bifidobacterium* vector-based oral cancer vaccine combination of anti-PD-1 and anti-CTLA-4 antibodies was enhanced in a mouse model of renal cell carcinoma [58].

Several clinical studies have proven the association between Bifidobacteria and the efficacy of tumor immunotherapy and the alleviation of adverse immune reactions. Oral live Bifidobacteria supplementation had a positive impact on patients undergoing colorectal cancer resection in terms of intestinal microbiota composition, immune system traits, and prognosis, according to a study by Zhang and colleagues [59]. Sixty patients were included in this study and randomly divided into two groups: the treatment group received enteral nutrition and oral live Bifidobacteria supplementation prior to surgery, and the control group received enteral nutrition only. Both preoperative and postoperative *Bifidobacterium/E.coli* (B/E) ratios in the control group (0.72 ± 0.14, 0.02 ± 0.06) were significantly lower than those in the treatment group (*p* < 0.05). On day 9 after surgery, the treatment group had higher fecal sIgA (secretory immunoglobulin A) levels and mucosal dendritic cell-mediated mucosal surfaces with anti-inflammatory properties. Zhang et al. also noted lower serum concentrations of IL-6, IgG, IgA, IgM, and CRP in the treatment group (*p* < 0.05). Additionally, compared to the control group, the therapy group experienced fewer postoperative septic problems. The number of additional hospital stays and hospital stay times were comparable. In addition to improving intestinal immunity and lowering postoperative problems, Bifidobacterial supplementation administered prior to colorectal cancer surgery may change the composition of the intestinal microbiota and help to restore its equilibrium [59].

In an open-label, single-center study (NCT03829111), 58% of metastatic renal cell carcinoma (RCC) patients received CBM588 (a live bacterial product containing *Clostridium butyricum*) combined with ICIs, compared with only ICIs treatment in 20% of patients. Furthermore, among patients treated with CBM588, PFS was significantly increased to 12.7 months compared to 2.5 months with ICIs alone [60]. Similarly, a preclinical and clinical investigation that generated a hypothesis implies that a combination of many commensal strains, including *Bifidobacterium*, also calls for additional clinical testing [46]. Nevertheless, despite the initial success of CBM588 as stated above, it is still unknown whether this whole strategy is feasible and effective [60]. Current oncology treatments are being combined with a number of experiments evaluating microbiome or targeted microbial methods, and the outcomes of these trials are highly anticipated [61]. Although the final results of these clinical trials have yet to be reported, this combination treatment strategy has shown significant efficacy in non-oncology diseases such as Clostridium difficile colitis and promises to have a clear advantage over FMT in optimizing long-term commitment to gut microbiota modulation in oncology treatment [61–63]. Overall, these findings suggest that *Bifidobacterium* may promote CTLA-4 blockade and PD-1/PD-L1, improving efficacy against ICIs and promoting tumor control.

Bifidobacteria not only show synergistic effects in combination with immune checkpoint inhibitors, but also play a positive role in reducing immune-induced adverse reactions. ICIs act on different organs throughout the body, and different organs may be targets for immune-mediated adverse reactions, with ulcerative colitis being one of the more common side effects. Although colitis and diarrhea have been recorded, PD-1 antibody therapy has fewer side effects than CTLA-4 antibody therapy. Ipilimumab, a CTLA4 antibody under development, has been linked to a variety of immune-mediated toxicities, including hepatitis, small bowel colitis, and skin reaction [63–65]. Drug-induced diarrhea (27–54%) and chronic colitis (8–22%) were linked to the use of ipilimumab and tremelimumab, two CTLA4 antibodies [66–70]. Therefore, researchers started studies to comprehend the involvement of the gut microbiota in the development of ulcerative colitis in patients receiving ipilimumab treatment. Researchers found that Bifidobacteria played a large role in improving the immunopathology associated with CTLA4 blockade but had no significant effect on the antitumor treatment itself [71]. The study established a mouse model of checkpoint blockade-associated autoimmunity by injection of anti-CTLA4 antibodies or isotype controls to test the response of mice to oral dextran sodium sulfate (DSS) and showed that disease was particularly severe in mice receiving 3% DSS and in mice receiving anti-CTLA4 antibodies. Histological sections of the colon of mice stained with hematoxylin and eosin showed increased proliferative inflammatory cell infiltration and ulceration and worse histopathological scores in the combination treatment group compared to the control group. The therapeutic effects of anti-CTLA4 antibodies in mice with melanoma were also observed in experiments in which data from colitis and tumor models were compared to mice receiving anti-CTLA4 antibodies similar to ipilimumab patients, consistent with clinical observations. Another study examined the effect of vancomycin in combination with DSS and anti-CTLA4 and showed that mice in the combination treatment group had significantly lower histopathology scores than those in the control group [72]. A study by Wang et al. revealed the important role of Bifidobacteria in reducing the incidence of colitis caused by CTLA4 blockade and found that vancomycin treatment enhanced the safety of colitis in mice treated with DSS + anti-CTLA-4. In addition, this study showed that *Bifidobacterium* reduced autoimmunity linked to anti-CTLA4 therapy and restored vancomycin-induced microbiota imbalance by downregulating several proteins such as KC, IL-6, and CFS3 [71]. Maruya et al. analyzed the role of PD-1 on the intestinal microbiota and discovered that PD-1^−/−^ mice with *Bifidobacterium* were significantly reduced. In addition, IgA synthesis improved the growth of beneficial and homeostasis-inducing microorganisms in the gut and offered defense against the emergence of autoimmune disorders [73].

*Bifidobacterium* is a genus of gram-positive bacteria that are rod-shaped, sometimes bifurcated at one end, single or arranged in a V-shape, fenestrated, stellate, nonacid-resistant, nonbudding, nonmotile, and exclusively anaerobic. Bifidobacteria inhibit tumor growth. This effect is achieved in three main ways: by activating the immune system, regulating the metabolism of intestinal probiotics to reduce the production of carcinogens, and inducing apoptosis in tumor cells, which hinders tumor development.

## Related mechanisms of Bifidobacteria in modulatory immunotherapy

The characteristics of *Bifidobacterium* that reduce the side effects of ICIs tumor immunotherapy and enhance the combined therapeutic effect of multiple immune checkpoint antibodies will help optimize relevant clinical immunotherapy protocols, laying a solid theoretical foundation for the use of probiotic strains to improve the structure of the intestinal probiotics and enhance the comprehensive effect of tumor immunotherapy [74].

**Fig. 2:**
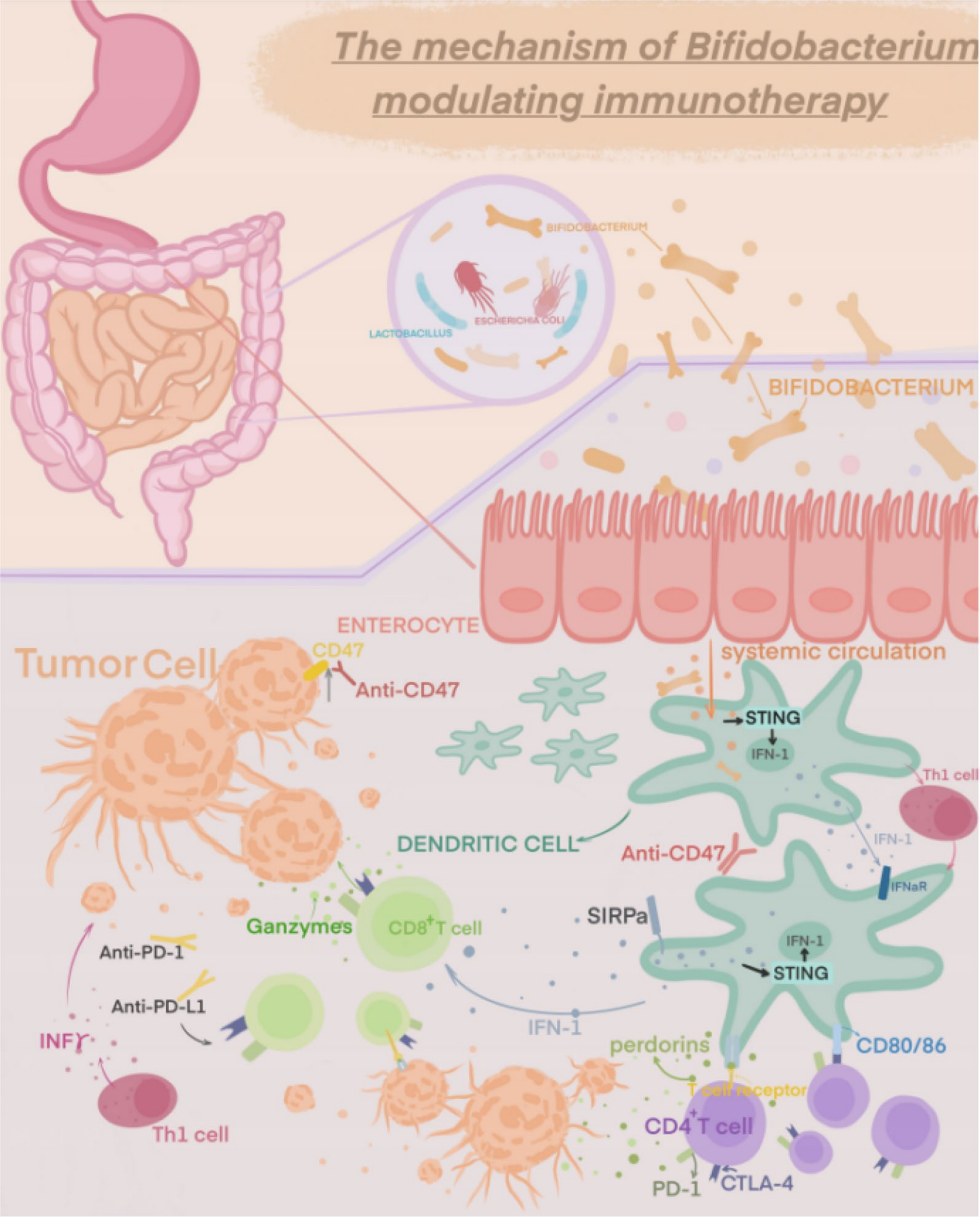
Modulatory effects of Bifidobacteria in cancer immunotherapy

The specific mechanism by which *Bifidobacterium* or other commensal bacteria stimulate anti-tumor immune responses is still unknown, despite the fact that it has been demonstrated that *Bifidobacterium* can enhance immunotherapeutic efficacy as a commensal organism capable of stimulating and modulating specific pathways through which it can influence the host’s innate and adaptive immune responses [43, 44, 75, 76]. Several studies have shown that *Bifidobacterium* modulates the immune function and inhibits tumor cell proliferation by possibly regulating different pathway mechanisms, among others [56, 77–79]. (Table 1) Several aspects of its possible mechanisms are described below (Fig. 2).

**Table 1:** Clinical and mechanistic advances in cancer associated with *Bifidobacterium* research

Major finding	Cancer/Therapy	Mouse/Human data	Classification	References
Microbiota-derived STING agonists induce intra-tumoral monocyte production of type I. IFN (IFN-I) to modulate macrophage polarization and NK-DC crosstalk	Melanoma/ICIs therapy	Mouse/Human	Microbiota-derived STING agonists and their effects on tumor immunity	[53]
Bifidobacteria promote local anti-CD47 immunotherapy in tumor tissue through their ability to accumulate in the tumor microenvironment. Systemic administration of *Bifidobacterium* leads to its accumulation within tumors and converts non-responsive mice into responders to anti-CD47 immunotherapy with stimulation of the interferon gene (STING) and interferon-dependent manner. Local delivery of *Bifidobacterium* was effective in stimulating STING signaling and increased cross-initiation of dendritic cells after anti-CD47 treatment	Colon cancer/Immunotherapy	Mouse		[77]
*Bifidobacterium longum (BL-Tum)* with pBBAD-Tum showed significant anti-tumor effects in tumor-bearing mice. The weight, volume, growth, MVD and vascular endothelial cell apoptosis of transplanted tumors in tumor-bearing mice treated with Tum-transformed *Bifidobacterium longum (BL-Tum)* were significantly lower than those in GFP-negative controls	Colon carcinoma	Mouse	Specific* Bifidobacterium* species and their effects on tumor growth, as well as their interactions with immunotherapy or checkpoint blockade	[80]
16S ribosomal RNA sequencing identified *Bifidobacterium bifidum* as being associated with antitumor effects. Oral bifidobacteria alone improved tumor control to the same extent as programmed cell death protein 1 ligand 1 (PD-L1)-specific antibody therapy (checkpoint blockade), whereas combination therapy virtually eliminated tumor growth	Melanoma/Immunotherapy	Mouse		[47]
Combination of *Bifidobacterium infantis* and adriamycin-supportive immunotherapy has a favorable anti-tumor effect on mouse mammary carcinoma	Breast cancer/Immune-combination therapy	Mouse		[50]
The bifidobacterial product CBM588 improves clinical outcomes in patients with metastatic renal cell carcinoma treated with nivolumab-ipilimumab	Metastatic renal cell carcinoma/nivolumab and ipilimumab with or without daily oral CBM588	Human		[60]
The efficient peripheral generation of antigen-specific populations of Treg cells in response to an individual’s microbiota provides important post-thymic education of the immune system to foreign antigens	N/A	Mouse	The role of microbiota in regulating Treg cell populations, influencing post-thymic education of the immune system, and modulating T-cell responses	[81]
Median progression-free survival (mPFS) was significantly longer in patients with *Bifidobacterium lactis* compared to patients without detectable *Bifidobacterium lactis* in their feces at baseline. The presence of *Bifidobacterium lactis* was an independently favorable prognostic factor influencing PFS in patients receiving combination therapy	Non-small cell lung cancer/anti-PD-1 immunotherapy combined with chemotherapy	Human		[52]
T cells targeting an epitope expressed in the commensal bacterium *Bifidobacterium breve* (B. breve) called SVYRYYGL (SVY) cross-reacted with the model neoantigen SIYRYYGL (SIY). Mice lacking *Bifidobacterium breve* had reduced SVY-reactive T cells compared to *Bifidobacterium breve*-colonized mice, and the T-cell responses could be shifted by SVY immunization or by co-housing* Bifidobacterium*-free mice with *Bifidobacterium*-colonized mice	Melanoma	Mouse		[82]
Intestinal *B. pseudogentian* modulates enhanced immunotherapeutic responses by producing the metabolite inosine. Immunotherapy resulted in reduced intestinal barrier function, which increased systemic transport of inosine and activated anti-tumor T cells	Immune checkpoint blockade (ICIs) therapy	Mouse	Influence of the intestinal microbiota, including specific* Bifidobacterium* species, on immunotherapeutic responses, tumor immunity, and the development of colon or renal cell carcinoma	[56]
*Bifidobacterium bifidum* rescued the immunopathology of mice to a large extent without a significant effect on antitumor immunity, an effect that may be dependent on regulatory T cells	Immunotherapy	Mouse		[71]
*Bifidobacterium infantis* (LBB) administration reduces tumor incidence, multiplicity/count and volume by enhancing TLR2-improved intestinal mucosal epithelial barrier integrity and inhibiting apoptosis and inflammation, thereby modulating the intestinal microbiota and reducing the development of colon cancer	Colon cancer	Mouse		[78]
*Bifidobacterium longum 420* significantly increased the survival of mice carrying renal cell carcinoma tumors treated with anti-PD-1 and anti-CTLA-4 antibodies compared to mice treated with antibodies alone	Renal cell carcinoma/anti-PD-1 and anti-CTLA-4 therapy	Mouse		[58]

### Promoting dendritic cell-dependent Th1 differentiation

Niers et al. [82] investigated the ability of probiotics to interact with neonatal dendritic cells (DCs) and identified the involvement of *Bifidobacterium bifidum* W23 in driving the initiation of Th1 and Tr1 responses in human neonatal DCs. A study by Mager et al. [56] used an animal model of colorectal cancer to identify bacteria that promote the efficacy of ICIs. The researchers found that *Bifidobacterium pseudolongum* provided the strongest facilitation of immune checkpoint blockade, and in the absence of ICI treatment, *Bifidobacterium pseudolongum* failed to induce anti-tumor immunity, but in small intestinal lamina propria CD4^+^ T cells, *Bifidobacterium* induced an increase in the expression of the Th1 master transcriptional regulator T-bet, suggesting that *B. pseudobifidum* has immunomodulatory capacity even in the absence of ICIs. Another research conducted by Li showed oral administration of *Bifidobacterium breve* (*B*. *breve*) lw01 could significantly up-regulate tumor cell apoptosis and inhibit tumor growth, which relied on the recruitment of DCs and tumor-infiltrating lymphocytes in tumor microenvironment. One of the mechanisms is the enhancement of interleukin 12 (IL-12) secretion derived from DCs is essential to *B. breve*’s antitumor effect, which was counteracted by the treatment of neutralizing antibody for IL-12 [83]. A more in-depth study of the ability of *B. pseudobifidum* to induce Th1 transcriptional differentiation during steady state and the mechanisms that activate Th1 effector function following treatment with ICIs revealed that the promotion of ICIs by *B. pseudobifidum* is mediated by inosine and is specifically dependent on adenosine 2A receptor (A2AR) signaling in T cells. The effect of inosine as a bacterial metabolite on T cells requires synergistic stimulation of DC (possibly via DCs), interleukin-12 (IL-12) receptors involved in Th1 differentiation and IFN-7 production for effective anti-tumor immunity. *Bifidobacterium pseudobifidum* enhances the role of ICIs in a mouse intestinal tumor model by enhancing conventional dendritic cell-dependent Th1 cell circuits. The immunomodulatory effects of *Bifidobacterium longum* KACC 91563 were investigated in mouse splenocytes and macrophages by Choi et al. This strain’s ability to control T and B cell proliferation was noted. Additionally, it prevented the proper balance of Th1/Th2 cytokines (i.e., Th2: IL-10, IL-4 and Th1: TNF-*α*, IL-2). IgE levels were increased following the use of *Bifidobacterium longum* KACC 91563. Therefore, this strain appears to have the ability to regulate the host immune system by producing IgE and acting by preserving and enhancing the Th1/Th2 balance [84]. The ability of three strains to modify known Th2 responses was evaluated by researchers in a tolerance induction model experiment in which *Bifidobacterium bifidum* NCC 453 promoted sublingual immunotherapy in a mouse model. They observed that these strains demonstrated polarized responses toward a Th1/Treg pattern and induced DC maturation [85]. *Bifidobacterium bifidum* can stimulate interleukin-12 (IL-12) production followed by enhanced recruitment of tumor-specific cytotoxic T lymphocytes (CTLs) and concomitant release of interferon-gamma (IFN-*γ*) [86, 87]. To clarify the many pathways connecting these signaling molecules, more research is needed.

### Enhancing CD8 + T cell activity

Sivan et al. [47] originally showed in animal studies that the gut microbiota might influence the tumor immune microenvironment because *Bifidobacterium bifidum* improved DC activity, which improved CD8^+^ T cell initiation and anti-PD-L1 efficacy. Leads to high percentage of CD8^+^ T cells, which contributes to the effectiveness of anti-PD-1 therapy [29, 57, 88].

*Bifidobacterium* was strongly linked to the accumulation of activated antigen-specific T cells in the tumor microenvironment [47]. *Bifidobacterium* was positively associated with delayed tumor growth and a good response to anti-PD-L1 treatment. High activation of tumor-specific T cells around the tumor and an increase in antigen-specific CD8^+^ T cells inside the tumor were both associated with considerably better tumor control in *Bifidobacterium*-treated mice compared to non-*Bifidobacterium*-treated mice. Supplementation with an oral probiotic containing *Bifidobacterium* spp. was also found to restore the anti-PD-L1 therapeutic effect in mice with “dysregulated” intestinal probiotics [89]. When investigating the mechanism of action, a significant expansion of CD8^+^ SIY-specific 2C T cells was observed in the tumor-draining lymph nodes of JAX mice and Tac mice treated with *Bifidobacterium*, as well as a significant production of IFN-7. This suggests an improved DC immune response upstream of T cells, consistent with an increased proportion of MHC-II hiDCs in the tumors of both mice. Examination of genome-wide transcripts of early tumor-infiltrating DCs in Tac mice, JAX mice and *Bifidobacterium*-treated Tac mice revealed that the expression of 760 genes was upregulated in DCs from JAX mice and *Bifidobacterium*-treated Tac mice, significantly enriched in cytokine–cytokine receptor interactions, T-cell activation and monocyte proliferation positive regulatory pathways, many of which are involved in co-stimulation [CD40, CD70, H2M2 (MHC-I) ICAM1] and CD8^+^ T cell activation, DC maturation (IFNGR2, RELB), antigen presentation and cross-presentation (TAPBP, RAB27A, SLC1 1A1), chemokine-mediated immune cell in the tumor microenvironment enrichment (CXCL9, CX3CL1, CXCR4), and type 1 IFN signaling pathway (IRF1, IFNAR2, OAS2, IFI35, IFITM1). DCs isolated from these 3 pups were found to induce proliferation of CD8^+^ T SIY-specific 2C T cells at lower antigen concentrations in DCs from Bifidobacteria-treated Tac mice and JAX mice compared to Tac mice. Moreover, DCs from JAX mice elevated IFN-7 production by T cells at any antigen concentration. This suggests that signaling produced by commensal *Bifidobacterium* can stably modulate DC activation, thereby improving the effector function of tumor-specific CD8^+^ T cells [47]. Subsequently, Matson et al. [29] examined stool specimens from metastatic melanoma patients prior to anti-PD-1 immunotherapy and found that stool samples from patients responding to anti-PD-1 treatment were enriched for *Bifidobacterium longum, Escherichia coli* and *Enterococcus faecalis*. Transferring the feces from patients to germ-free mice showed that mice receiving feces from patients who had responded to anti-PD-1 treatment had slower tumor growth and significantly higher efficacy of anti-PD-L1 immunotherapy compared to mice receiving feces from patients who had not responded to anti-PD-1 treatment. The effect was mediated by an increase in CD8^+^ T cells and a decrease in FoxP3^+^ Treg cells in the tumor microenvironment. This approach was found to inhibit tumor growth in a homozygous mouse bladder cancer model using a *Bifidobacterium* vector-based oral cancer vaccine. Intercellular cytokine staining (ICCS) data suggest that *Bifidobacterium longum* 420 induces activated CD8^+^ and CD4^+^ T cells in the spleen and MLN and may activate dendritic cells (DCs) and Peyer ‘s patches (PPs) in mesenteric lymph nodes (MLNs) [90]. Furthermore, a recent study revealed that molecular mimicry between *Bifidobacterium*-derived epitopes and melanoma may favor activation of cross-reactive T cells and constitutes one of the mechanisms by which gut microbiota affects antitumor efficacy and modulates OVs response [91].

CD47 is a signaling molecule that helps tumor cells escape the immune system, and it has been found that blocking CD47 can have a beneficial effect on tumor immunotherapy [56, 92]. Researchers have shown that intestinal probiotics can further stimulate the body to produce anti-CD47 antibodies by activating the STING signaling pathway [56]. Experimentally, wild-type (WT) mice, antibiotic-fed mice and germ-free (GF) mice from different institutions differed in their immune anti-tumor effects against CD47. GF mice differed in their immune antitumor effects against CD47. The anti-tumor response to CD47 blockade treatment in non-responding mice (Tac mice) was restored by contact transmission or oral transfer of commensal bacteria from responding Jax mice through mouse cohabitation. Subsequent results from *Bifidobacterium* injections showed antitumor efficacy in mice that did not respond to anti-CD47 treatment. It suggests the tumor-targeting ability of *Bifidobacterium* is a possible mechanism by which the intestinal probiotics affects the antitumor response. The antitumor effects of CD47 blockers are dependent on type I IFN signaling and cross-initiation of tumor-associated DCs. I-IFN receptor-blocking antibody to Tac mice and discovered that blocking type I IFN signaling prevented the administration of anti-CD47 therapy even in the presence of *Bifidobacterium*, highlighting the significance of type I IFN signaling in DCs for the treatment of CD47 blockade caused by Bifidobacteria. Subsequently, to observe the effect of *Bifidobacterium* on type I IFN signaling in DCs, researchers assessed IFN-P expression in isolated tumor DCs. IFN-P expression was increased in DCs from Tac mice treated with *Bifidobacterium* and anti-CD47, but not in mice treated with anti-CD47. Type I IFN signaling promotes the cross-initiation of DCs and stimulates adaptive immune responses [93, 94]. The STING pathway regulates type I IFN expression in anti-CD47-mediated antitumor effects [56, 94]. However, it is still unclear how the STING pathway is related to *Bifidobacterium*’s antitumor activity. In Tmeml73-/- mice with the STING gene knocked down, it was found that the use of *Bifidobacterium* failed to support anti-CD47 immunotherapy. As a result, the STING signaling pathway is essential for Bifidobacteria’s antitumor actions [56]. Specific members of the intestinal probiotics can enhance the antitumor efficacy of immunotherapy by colonizing tumor sites. An anaerobic commensal bacterium found in the gastrointestinal tract, *Bifidobacterium*, has the ability to infiltrate the interior of tumors and control the immune response induced by CD47 inhibition. *Bifidobacterium bifidum* has the potential to be an effective clinical target for tumors, given its characteristics [56].

### Inducing immune responses against microbial antigens that cross-react with tumor-associated antigens

A related study found that *B*. *bifidum* PRL2010 sortase-dependent bacterial hairs inhibited the expression of additional pro-inflammatory cytokines linked to systemic responses, such as IL-12, while activating a variety of signals in macrophages by locally producing high amounts of the cytokine TNF-. Apparently, this facilitated a close association between the host immune cells and *Bifidobacterium* strain [95]. This may be an important aspect of Bifidobacterial regulation of immunity that warrants further investigation. SVYRYYGL(SVY) peptides placed on mouse MHC (Major Histocompatibility Complex) bound to a model TCR (T-cell receptor) specific for the KbSIY complex, but in a different conformation, according to investigations using molecular dynamics simulations by Catherine A. Bessell et al. With a preference for either SIY or SVY, heterozygous CD8^+^ T cell populations grown from wild-type C57BL/6 mice reacted cross-reactively with bacterial and tumor antigens. One of their study’s most intriguing conclusions was that the gut microbiota induced T-cell responses that were SVYRYYGL (SVY)-reactive. Analysis of mice with and without *Bifidobacterium* demonstrated that in vivo initiation occurred and improved the ability to increase SVY-reactive T cells, even though the mechanisms of T cell activation and selection by components of the gut microbiota remain unknown. Due to their immunogenicity, T cells targeting an epitope called SVYRYYGL (SVY) expressed in the symbiotic bacterium *Bifidobacterium breve* cross-react with the model neoantigen SIYRYYGL (SIY). T-cell responses that are specific for an antigen are also metastable [96]. Laurence Zitvogel and Guido Kroemer summarize evidence that microbial/tumor cross-reactive immune responses are clinically relevant, particularly when the microbes are part of the gut microbiota. The gut microbiota affects host physiology at multiple levels, reflecting the complexity of ecosystem changes in which microbial species may influence immune tone by acting on metabolic pathways, neuroendocrine circuits, and pathogen recognition receptors [97].

Currently, it is uncertain how T cell populations are affected by homologous epitopes between tumors and the microbiota. As a result, mechanistic studies must focus on how cross-reactive T-cell populations react to tumor cells and how bacterial colonization affects cross-reactive T-cell populations to gain insight into the antigens expressed by the microbiota and their potential cross-reactivity with T cells that recognize tumor-specific neoantigens.

The regulatory mechanisms of Bifidobacteria in cancer immunotherapy involve three main aspects: In the tumor immune microenvironment,* Bifidobacterium intestinalis* promotes dendritic cell-dependent Th1 differentiation; enhances CD8^+^ T cell activity to modulate the immune response; and induces immune responses against microbial antigens that cross-react with tumor-associated antigens.

## Summary and outlook

Antitumor medications and the gut microbiota interact in a very complex way through pharmacodynamics and pharmacokinetics (e.g., enzymatic degradation and metabolism). At the same time, tumor immunotherapy can alter the composition of the microbiota, producing bidirectional interactions [98]. Bifidobacteria have been shown to selectively accumulate in tumors upon entry into the blood stream, possibly due to the hypoxic microenvironment’s eosinophilic nature and immunosuppressive conditions for bacterial colonization and proliferation [47]. Bifidobacteria improved anti-cancer immune monitoring and increased the abundance of tumor-infiltrating CD8^+^ T cells [47]. Thus, *Bifidobacterium* is considered a promising delivery vehicle for tumor-specific transport [99]. In future bioengineering research, genetic improvement of *Bifidobacterium* strains and strengthening the development and exploitation of *Bifidobacterium* preparations will provide new ideas and methods for future tumor research and tumor treatment.

Bifidobacteria have been shown to increase efficiency and reduce side effects in tumor immunotherapy, but the underlying mechanisms of action are unclear. Understanding the relationship between Bifidobacteria and immunotherapy is gaining popularity because it may have a substantial impact on the effectiveness and safety of some anti-cancer medications as well as the creation of effective, individualized clinical therapies. Most of the current findings are derived from studies conducted in animal models, and further research should focus on clinical combinations with other antitumor therapeutic modalities as well as on the impact of *Bifidobacterium bifidum* in combination with ICIs on overall cancer survival and on interactions with other treatments. The effect of *Bifidobacterium* on host immunity is considered one of the important factors influencing antitumor immunity and therapeutic efficacy. The current clinical approach of using microbial agents with immunotherapeutic effects (e.g., probiotic strains such as *Bifidobacterium bifidum*) to enhance the efficacy of cancer immunotherapy is still in its infancy, and in future research, a deeper understanding of the mechanisms of microbial modulation of immunity and identification of immunostimulatory and immunosuppressive related bacterial strains could improve the feasibility and efficacy of therapeutic approaches, thereby increasing tumor cure rates [100].

The rapid development of tumor immunity has brought new light to the cure of tumors, and as research continues, the understanding of the mechanisms of tumor development and the complex relationship between its organism’s immune system is becoming clearer. Immunotherapy holds promise as an ideal treatment for cancer patients. At this stage, more standardized and unbiased pre-clinical and clinical studies are needed to ensure that the treatment effect is sufficiently reliable and significant in the bidirectional relationship between immunotherapy and the human gut microbiota in cancer patients. The study of gut probiotics has benefited many patients, but as more and more research progresses, researchers are noticing that tumor immunotherapy is not a panacea, as there are still a large number of studies finding poor response, slow effect, drug resistance, and other problems after the organism has been given immunotherapy. Therefore, how to refine the therapeutic regimen of tumor immunity by modulating or intervening in tumor immunity to achieve greater clinical benefit for patients is unclear. This is an important direction for basic and clinical research.

Although Bifidobacteria open up a new avenue for tumor immunotherapy, there are still many challenges and limitations to the clinical applications that remain to be realized. This is because the ability of *Bifidobacterium* to be used directly as a biomarker for colorectal carcinomatosis still needs further clarification. An international standardization of methods for intestinal cohort collection and sequencing analysis is needed. Further research is still required for the synergistic development of basic and clinical studies to facilitate clinical translation.

The relationship between the gut microbiome and tumor immunotherapy is extremely complex. It is unclear which component of *Bifidobacterium* is the most ideal focus for tumor immunotherapy. Therefore, there is a need to develop multiple research programs in future clinical studies, such as fecal microbiome transplantation studies and dietary studies. Despite the important role of Bifidobacteria in tumor immunotherapy, the exact mechanism by which they trigger the immune response has not been fully investigated. Currently, most studies aimed at linking Bifidobacteria to immunomodulatory effects have been conducted using cell lines/animal models and therefore do not provide a comprehensive understanding of all the effects that Bifidobacteria have on the human immune system. Future research challenges therefore require more robust clinical trials to better understand the relationship between Bifidobacteria and tumor immunotherapy and the mechanisms underlying.
